# Fungicidal and plant growth-promoting traits of *Lasiodiplodia pseudotheobromae*, an endophyte from *Andrographis paniculata*

**DOI:** 10.3389/fpls.2023.1125630

**Published:** 2023-06-14

**Authors:** Gayathri Segaran, Mythili Sathiavelu

**Affiliations:** School of Biosciences and Technology, Vellore Institute of Technology, Vellore, Tamilnadu, India

**Keywords:** agriculture, biocontrol agent, chemical pesticide, endophyte, food production, phytopathogenic fungi

## Abstract

**Introdution:**

Fungal endophytes are widespread and dwell inside plant cells for at least part of their life without producing any symptoms of infection. Distinct host plants may have different fungal endophyte rates and community compositions. Despite this, the endophytic fungi connected with the host plant and their hostile behaviors, remain unknown.

**Methods:**

The objective of the current research was to isolate and identify endophytic fungal species from the root of *Andrographis paniculata*. The effects of fungal isolate APR5 on the mycelial growth of phytopathogens and the production of plant-promoting traits were assessed.

**Results and discussion:**

Endophytic fungal isolate APR5 showed higher inhibitory efficacy in dual and double plate assay against the tested phytopathogenic fungi. The scanning electron microscope analysis demonstrated that the phytopathogenic fungal hyphae were coiled by endophytes which makes them shrink and disintegrate. Further, an ethyl acetate crude extract effectively suppressed the mycelium growth of *Rhizoctonia solani* by 75 ± 0.1% in an agar well diffusion assay. The fungal isolate APR5 was identified as *Lasiodiplodia pseudotheobromae* using the nuclear ribosomal DNA internal transcribed spacer (ITS) region and qualitatively evaluated for their capacity to produce plant growth-promoting hormones. Gas chromatography-mass spectrometry was implemented to acquire a preliminary understanding of the secondary metabolic profile of ethyl acetate crude extract. 1-octadecene, erythritol, niacin, oleic acid, phenol, pantolactone, phenyl ethyl alcohol, *p*-cresol, and tbutyl hydroquinone are the metabolites analyzed in a crude extract of APR5 isolate and are reported to have antimicrobial properties.

## 1 Introduction

The global population has grown significantly from 1.6 billion in 1900 to 7.0 billion in 2011, in the past century. It is estimated that by the year 2050, there will be 9.7 billion people around the world, increasing the demand for water resources. Under this scenario, the production of food will need to expand by around 70% by 2050 and twice or triple by 2100, while aiming to reduce the impact on the environment (Poveda et al., 2021). Fungi are a prominent disease-causing agent on plants with a huge loss of up to 90% of agricultural production (Elamathi and Mathanraj, 2017). Soil-borne fungal pathogens reduce agricultural productivity and degrade the quality of food products. Such fungal infections with a wide host range spread diseases in a variety of commercially important crops (Dukare et al., 2020). These well-known soil-borne pathogens may be found in many types of soil. Due to their saprophytic nature, they may spend more time in the soil. This condition has been documented in at least 32 nations, with warm-climate countries being the hardest impacted (Karthika et al., 2020). Banana, cucumber, potato, tomato, and tobacco are the mainly affected crops by soil-borne pathogens all over the globe. The deadliest ailment to strike tomato plants worldwide, particularly in uplands, is Fusarium wilt. In the wilted plants with yellowed leaves, Fusarium wilt causes a 60–70% reduction in fruit output and infects 30–40% of the crop annually (Jinal and Amaresan, 2020; Karthika et al., 2020).

*Macrophomina phaseolina* causes seedling blight, charcoal, stem, and root rot and affects approximately 500 plant species from over 100 families all around the world. It affects commercially significant vegetables, cotton, sorghum, sunflower, and legumes and has a wide geographic spread in tropical and subtropical nations. When exposed to humans, *M. phaseolina* can infect immunosuppressed patients (Javed et al., 2021). When the temperature is high (30–35°C) and soil moisture is low (under 60%), it lowers farmer profitability by inducing major yield loss in sorghum and soybean. When the disease emerged at the pre-emergence stage, groundnut cultivars experienced 100% yield loss (Marquez et al., 2021). Due to its enduring nature, it can survive for up to 3 years in the shape of microsclerotia as resistant forms in infected plant detritus or dirt (Khan et al., 2021). *Rhizoctonia solani* is a major soil-borne fungus detected in both cultivated and non-cultured soils. It lives as sclerotia in the soil and does not produce asexual spores. The most prevalent infection induced by *R. solani* is seedling damping-off (Goudjal et al., 2014). Sclerotia are superficial, firm, and distinctively shaped dark brown to black masses, are the most obvious symptom of black scruf, and result in distorted and fractured tubers. The wide host range and overwintering characteristics of *R. solani* make them difficult to control using conventional biological and chemical methods (Rafiq et al., 2020).

To suppress the occurrence of soil pathogenic fungi, synthetic fungicides notably bavistin, benomyl, and thiram have traditionally been utilized (Dukare et al., 2020). These fungicides were transformed into poisonous compounds by the host plant tissue or by pathogens. In addition to fungicide resistance and increasing soil pollution, the widespread use of chemical fungicides has the potential to disrupt microbial ecosystems and weaken the ozone layer (Goudjal et al., 2014). About 10 to 40% of the nutrients from chemical fertilizers are ultimately absorbed by plants and the remainder are leached, their use would aid in reducing the loss of nutrients (Poveda et al., 2021). The rise in production demand, restrictions on agrochemicals usage, and the emergence of resistance towards the chemical products used led to the need for new and effective biocontrol agents (Elamathi and Mathanraj, 2017). Due to their non-polluting and eco-safe nature, biocontrol agents with plant growth-promoting traits can leads to chemical-free sustainable agriculture (Dukare et al., 2020).

Endophytic fungi are ubiquitous and stay intercellular or intracellular in plants for at least a portion of their lives without triggering infection symptoms (Nayak et al., 2016). Darnal, Germany discovers endophytes in 1904. Endophytic fungi similarly colonize plant tissues as plant pathogens and mycorrhizae, with a series of stages that include host recognition, fungal spore germination, epidermal penetration, and tissue colonization (Nayak et al., 2016). Mutualistic, symbiotic, communalistic, and trophobiotic are the various interaction types found between host plants and endophytes (Masi et al., 2019). The frequencies and community compositions of fungal endophytes may vary for different host plants (Piska et al., 2015). Endophytic fungi are identified to have mutualistic relationships with their hosts and mostly protect plants from tissue-invading pathogens or herbivores by producing secondary metabolites, phytohormones that encourage plant development, or by delivering nutrients to the host. They may also interact directly with their hosts through niche competition, hyperparasitism, by releasing poisonous substances and by inducing systemic resistance (Radu and Kqueen, 2002; Bila´nski and Kowalski, 2022). By secreting plant growth-promoting chemicals that might confer resistance to the host plant during favorable environmental circumstances, the endophytic fungi improve the growth response in infected host plants mostly through nutrient cycle (Piska et al., 2015). Endophytic microorganisms are a relatively unexplored community that is currently gaining attraction in medical and agricultural research. Different researchers worked on the endophytic fungi of various medicinal plants in and around India (Nayak et al., 2016; Roy et al., 2016).

*Andrographis paniculata* is an erect annual herb with a harsh flavor and belongs to the Acanthaceae family. Andrographis is a genus of little annual shrubs with 28 species primarily found in tropical Asia. In north-eastern India, the plant is known as Maha-tita, or “king of bitters” (Nayak, 2015). It is native to India and Sri Lanka and the plant is extensively cultivated in Asia. In China, Indonesia, Hong Kong, the Philippines, Malaysia, and Thailand, it is used as traditional herbal medicine. It is referred to as Hempedu Bumi in Malaysia. Flavonoids, diterpenes, lactones, aldehydes, alkanes, and ketones were found in this medicinal plant. Andrographolide and Kalmeghin are the bioactive chemical compounds found in their leaves (Firdous et al., 2020). In addition to its widespread usage as an immunostimulant, Kalmegh is stated to have anti-snake venom, antihepatotoxic, antimalarial, antibiotic, antihepatitic, anti-inflammatory, antipyretic and anti-thrombogenic effects (Nayak, 2015). It is recognized as ‘Sirunangai’ or ‘Siriyanangai’ in Tamil (Arunachalam and Gayathri, 2010). *A. paniculata* harbors endophytic bacteria with the capability to act as plant growth regulators and promoters (Masi et al., 2019). 14-Deoxyandrographolide, 14-deoxy-11,12-didehydroandrographolide, neoandrographolide, and andrographolide are the labdane type diterpene lactones and a major bitter component of this plant (Rashid et al., 2018). Due to the existence of many bioactive metabolites, medicinal plants have a specific microbiome that can enhance the potential for interaction with microorganisms. Plant growth-promoting microbes stimulate plant growth by competing with microbial pathogens, activating plant defense responses, and secreting plant growth-promoting chemicals that include auxins, bacterial volatiles, and cytokinins (Sinha and Raghuwanshi, 2015). Research findings on medicinal plants and endophytes have revealed that the therapeutic properties of medicinal plants are not only due to the chemicals found in the plant but also to the endophytes that dwell within the plant (D’Souza and Hiremath, 2015). Selecting plants for investigating endophytic fauna for a specific goal usually involves several considerations. The objective of this experiment was to evaluate the hostile behavior of soil-borne pathogenic fungi and fungus endophytes from the plant *A. paniculata*. The present research will contribute to the investigation and application of endophytic fungus for enhanced plant disease management.

## 2 Materials and methods

### 2.1 Procurement of indicator microorganisms

*Fusarium oxysporum, Macrophomina phaseolina*, and *Rhizoctonia solani* are the diagnostic phytopathogens used for assessing the antifungal ability of endophytic fungus. The Department of Plant Pathology at Annamalai University in Chidambaram graciously provided with these soil-borne fungus phytopathogens. The maintenance and cultivation of fungal strains were carried out on a potato dextrose agar (PDA, HiMedia Laboratories, Mumbai, India).

### 2.2 Isolation of endophytic fungus from *Andrographis paniculata*

The root segments were carefully detached from the healthy *Andrographis paniculata* in Ranipet district, Tamilnadu, India (Latitude 12.9272; Longitude 79.36883). The plant parts were cleaned with distilled water to get rid of dirt and debris. Following 4% NaOCl solution for 3 minutes, 70% ethanol for 1 minute, and 70% ethanol for 30 seconds, the surface of the root was sterilized. Upon that, the root segments were washed three times with clean Milli-Q water. 100 µL of Milli-Q water from the final wash was spread over the fresh PDA plate to check the efficacy of surface sterilization (control plate). The surface-sterilized root segments were then cut into tiny sections of about 0.5 cm, placed on PDA plates, and incubated for 7 to 10 days at 27 ± 2°C until the fungal endophytes appeared. The fungal strains were purified using the single hyphal tip method and then plated on a PDA medium (Rakshith et al., 2013).

### 2.3 Antagonistic activity

#### 2.3.1 Dual culture method

A dual culture experiment was conducted to evaluate the antagonistic activity of endophytic fungus against soil-borne phytopathogens. On the opposing plate, active pathogenic (3 days old culture of *F. oxysporum, M. phaseolina*, and *R. solani)* and endophytic fungus were put as 8 mm mycelial plugs with a 3 cm gap between them and 1 cm from the border. The control dish contains only the pathogenic fungi disc. The experiment was carried out in triplicates and incubated at 27 ± 2°C. When the pathogenic fungus completely covered the control dish, growth suppression was recorded (Rakshith et al., 2013).

The following equation was used to estimate the growth inhibition rate:

The percentage of inhibition (%) = [(RC−RT)/RC] ×100

Whereas RC denotes the radius of the control colony,

RT denotes the radius of the test colony.

#### 2.3.2 Double plate technique

Endophytic fungus were grown on sealed petri plates to assess their volatile compound production. About 5 mm discs of test pathogens and endophytic fungi were each placed in the center of two separate bottom petri dishes. One of the plates (with the pathogen) was then flipped over to the other bottom containing endophyte to form a chamber. This experimental setup was sealed with parafilm and kept for 7 days at 27 ± 2°C. Endophytic fungus without pathogens at the bottom were used as a control. The percentage of inhibition was assessed following a week of monitoring (Chen et al., 2016).

#### 2.3.4 Scanning electron microscopy analysis

Visualization of the morphologic changes in pathogenic fungus was done using scanning electron microscopy (SEM) analysis. To investigate changes in the hyphal morphology of test fungi caused by the antagonistic action of endophyte, 0.5 cm pieces of agar media from the edge of the inhibition zone were analyzed. The samples were prepared to view under SEM (EVO/18 Research , Carl Zeiss).

### 2.4 Molecular genomic identification of the endophytic fungus

Molecular identification was carried out by employing 18S rRNA sequencing. Using the NucleoSpin^®^ Tissue Kit, the genomic DNA was extracted. Using the universal primers ITS-1F (5’-TCCGTAGGTGAACCTTGCGG-3’) and ITS-4R (5’-TCCTCCGCTTATTGATATGC-3’), the genomic DNA was amplified. The PCR was conducted in a 20 µL reaction mixture that comprised 5 pM of forward and reverse primers, template DNA, 0.1 mg/mL BSA, 1 unit of AmpliTaq Gold DNA polymerase enzyme, 1X PCR buffer (100 mM Tris HCl, pH-8.3; 500 mM KCl), 0.2 mM each dNTP (dATP, dGTP, dCTP, and dTTP) and 2.5 mM MgCl_2_. The 40 cycles of denaturation at 95°C for 30 s, annealing at 58°C for 40 s, extension at 72°C for 60 s, and final extension at 72°C for 5 minutes were the first step in the amplification process. A PCR thermal cycler was used to do the PCR amplification (GeneAmp PCR System 9700, Applied Biosystems). The PCR results were examined using a UV transilluminator and 1.2% agarose gel electrophoresis. Using BLAST search, the outcomes were matched to the National Center for Biotechnology Information (NCBI). A phylogenetic tree was created by the neighbor tree joining method (Jenila and Gnanadoss, 2018).

### 2.5 Analysis of the antifungal activity of a crude extract

#### 2.5.1 Preparation of endophytic fungal extracts

The endophytic fungus (3 days old culture) were grown for 21 days under steady circumstances at 27 ± 2°C in a 500 mL conical flask (Borosil graduated narrow mouth flasks code 4980024) containing 300 mL potato dextrose broth. After the incubation period, Whatman No. 1 filter paper was deployed to separate the fungal mat from the culture filtrate. Multiple solvents with different polarities were used to extract the secondary metabolites from culture filtrates using a separating funnel (Borosil funnel code- 6400). Petroleum ether (pet ether), dichloromethane (DCM), ethyl acetate (EA), and butanol were some of the solvents utilized. The chemical compounds were extracted from the fungal mycelium mat using methanol. The organic phase was obtained and condensed in a rotary evaporator (Model: RE100-Pro). The extracted metabolites were dried and stored for further analysis at -20°C.

#### 2.5.2 Agar well diffusion assay

Phytopathogenic fungal discs of about 8 mm (*F. oxysporum, M. phaseolina*, and *R. solani)* were placed in the center of a fresh PDA plate, and different solvent extracts of various filtrate concentrations (25, 50, 75, and 100 µg/mL) were loaded into four wells made in equivalent distance. For antifungal tests, the desiccated crude preparations were reconstituted with dimethyl sulfoxide (DMSO). The control plates with pathogens were loaded with 10% DMSO solvent and incubated at 27 ± 2°C. The results from the control plate were compared to the proportion of mycelial growth inhibition in the test plate. Three replications of the assay were done for each treatment. Using the above-mentioned formula, the growth inhibition percentage of phytopathogens’ radial mycelial growth was calculated (Wei et al., 2020).

#### 2.5.3 Poisoned food technique

Poisoned food bioassay was used to evaluate the effectiveness of fungal crude extracts against phytopathogens. Molten PDA medium was mixed with fungus extracts (1000 µg, 500 µg, and 250 µg/mL DMSO), which are thought to be poisonously feeding for pathogens. Intoxicated PDA plates were inoculated with a pathogen’s mycelia plug, which was then incubated at 27 ± 2°C for 7 days. By contrasting the radial expansion of the pathogen cultured in the test and control plates (DMSO), the impact of extracts on the growth of the pathogen was identified. The inhibition percentage formula was used to calculate the findings as a percent suppression of pathogen development (Gupta et al., 2022).

#### 2.5.3 GC-MS analysis

GCMS was used to assess the crude extract of *L. pseudotheobromae* APR5. The investigation was done on an Agilent 7890B gas chromatography system and an Agilent MS 240 Ion Trap with HP-5MS capillary column (5 percent phenyl methyl polysiloxane, 30 m, 250 M, 0.25 M). The startup oven temperature was 50°C, which was set for 1 minute, proceeded by a 10°C min^-1^ ramp to 200°C, which was held for 1 minute, then a 5°C min^-1^ ramp to 325°C, which was held for 1 minute. A total of 1 liter was supplied, and the temperature was maintained at 280°C. The carrier gas was helium, and the ionizing electron energy was 70 eV. The extract was separated into tenths of a liter. The ions were found in the 50–1000 m/z range. The GC required 25 minutes to complete. The dried crude obtained was diluted with the same solvent and studied with GC-MS analysis (Veilumuthu et al., 2022).

#### 2.5.4 Analysis of ethyl acetate crude extracts by Fourier transform infrared spectroscopy

The fungal crude extracts were FTIR analyzed using a Shimadzu FT-IR spectrophotometer (Model: IR Affinity). The functional groups contained in the chemical compounds were recorded in the range of 4000–400 cm-1. The infrared absorption spectrum is used to determine the chemical bonds in the molecule. The annotated spectrum indicates that the chemical bonds in the sample absorb a certain wavelength of light. For FTIR instrumentation examination, the dried crude extract of fungus was employed (Veilumuthu and Christopher, 2022).

### 2.6 Plant growth-promoting traits

For the screening of indole acetic acid (IAA) synthesis, the isolated endophyte was grown on Czapek broth medium for 7 days at 27 ± 2°C. After seven days, the samples were filtered, and the amount of IAA in the culture filtrate was measured by the addition of 1 mL of Salkowski reagent to 2 mL of the filtrate and incubated for 30 minutes in the dark (Bilal et al., 2018). To determine whether siderophores were present, 1 mL of the fungus culture’s supernatant was combined with 0.4 mL of 2% liquid FeCl_3_. The transition from yellow to brown confirms the presence of siderophore synthesis. To investigate the production of hydrocyanic acid (HCN), Whatman paper strips (dipped in the solution of 0.3% picric acid and 1.5% Na_2_CO_3_) were attached to the top lid of a petri dish, and fungus isolates were grown on a PDA medium. When the yellow color paper strip turns brown, it is considered to be positive. The presence of ammonia generation was detected by adding 2–3 droplets of Nessler’s reagent to the culture supernatant of fungus grown in 10 mL of peptone. Pikovskaya’s agar medium (PVK, Himedia) was supplemented with 0.1% zinc oxide and 2.5% tricalcium phosphate at pH 7.0 ± 0.2 to screen the ability of the fungal isolate to solubilize phosphate. The inoculation of fungal culture was done on a medium and after 24–48 h of incubation at 28°C, the formation of a halo inhibitory zone around the fungal radial growth indicated a positive outcome (Chowdhary and Sharma, 2020).

### 2.7 Statistical analysis

To achieve three values for *in vitro* experiments, samples were analyzed in three replicates and the outcomes were measured. The GraphPad Prism Version 9.5.1 (733) software was used to perform the statistical analysis. All results were presented in terms of mean ± standard deviation (SD).

## 3 Results

### 3.1 Ethnomedicinal investigation of selected medicinal plants

In the present research, *Andrographis paniculata*, a medicinal plant was examined for its fungal endophytes. Being one of the bitterest herbs, it is highly valued in traditional medicine. Previous studies documented the anti-fungal and anti-typhoid properties of plant extracts. Consequently, the primary goal of this work was to identify a potent endophytic fungus with biocontrol and plant growth-promoting traits.

### 3.2 Isolation and identification of fungal endophytes from *Andrographis paniculata*

**Figure 1:**
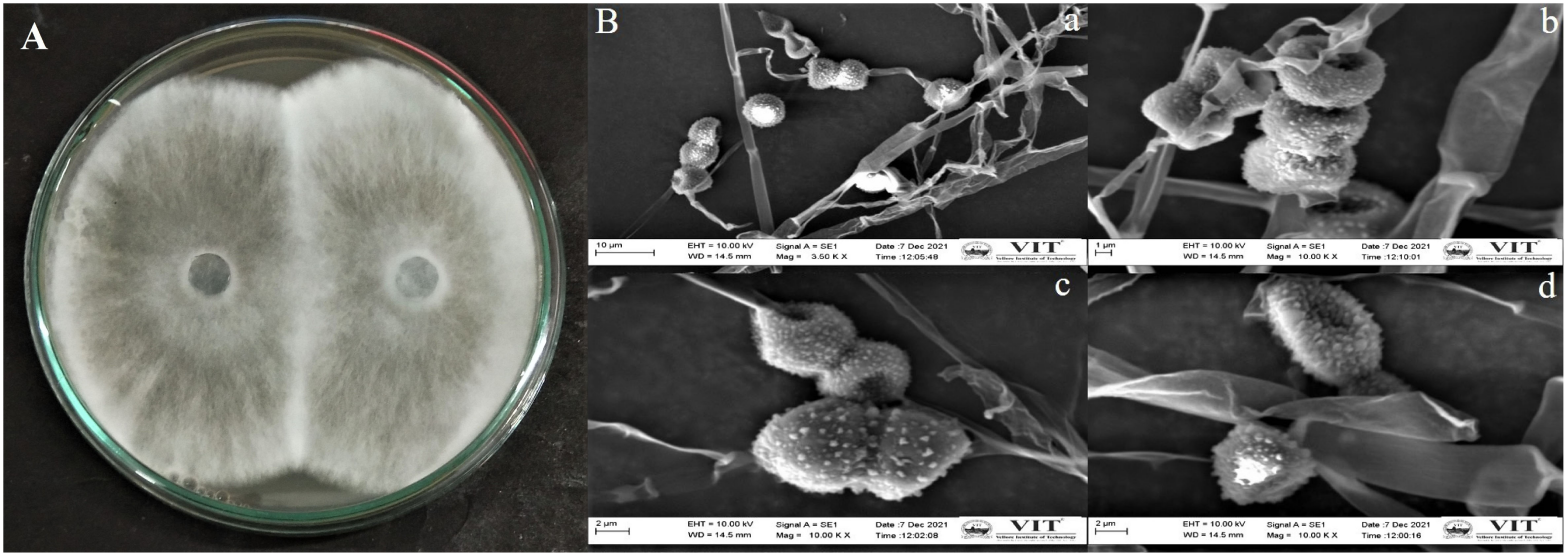
**(A)** Morphological appearance of endophytic fungus *Lasiodiplodia pseudotheobromae* APR5 isolated from *Andrographis paniculata* root; **(B)** The scanning electron micrographs of spores and hyphae of endophytic fungus *Lasiodiplodia pseudotheobromae* APR5 **(A)** (scale bar = 10 μm), **(B)** (scale bar = 1 μm), **(C)** (scale bar = 2 μm), **(D)** Morphological characteristics of a single spore (scale bar = 2 μm). The spores and hyphae were observed at 3500x and 10,000x.

**Figure 2:**
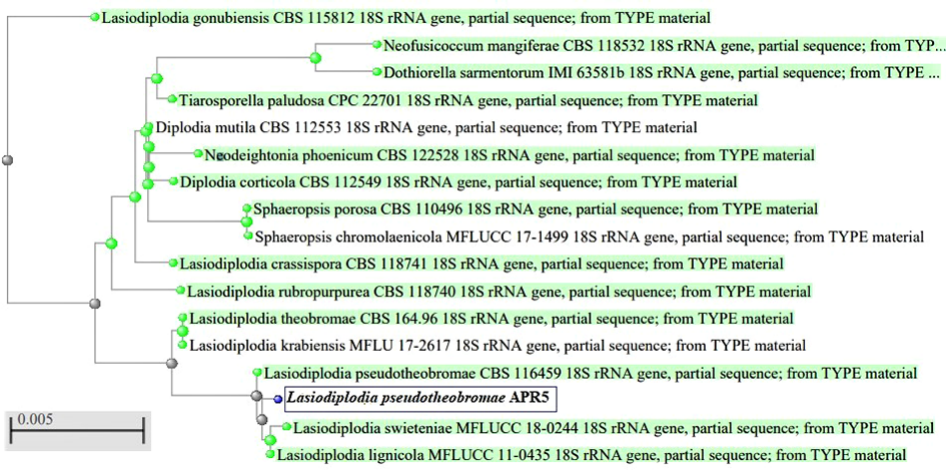
Phylogenetic tree of endophytic fungus *Lasiodiplodia pseudotheobromae* APR5 isolated from the root region of *Andrographis paniculata*.

The endophytic fungus isolate APR5 was isolated from the healthy roots of *Andrographis paniculata*. The single hyphal tip approach was used to purify the endophytic fungal isolates, which were then plated on a PDA medium (**Figure 1**). No fungal or bacterial growth was observed on the control plates. The fungal isolate APR5 is a fast-growing white fungus that turns black after 72 h. The results of the ITS analysis showed that isolate APR5 was most similar to *L. pseudotheobromae* with >97% identity. The 18S rRNA sequence of *L. pseudotheobromae* isolate APR5 was deposited in Genbank and the accession number (OP999617) was received. The sequences from the nucleotide BLAST result were used to create the phylogenetic tree (**Figure 2**). To our knowledge, no studies have been conducted on the biocontrol ability and plant growth promoting traits of endophytic fungal isolate *L. pseudotheobromae*.

### 3.3 The inhibitory effects on phytopathogenic fungi

#### 3.3.1 Antagonistic activity of endophytic fungus on test pathogens

**Figure 3:**
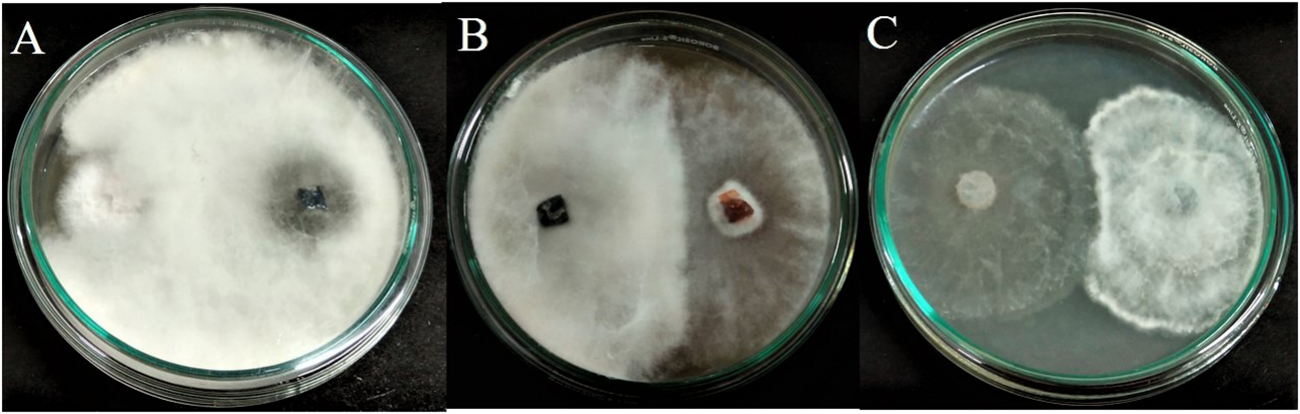
Antagonistic activity of endophytic fungus *Lasiodiplodia pseudotheobromae* APR5 towards tested soilborne phytopathogens **(A)** *Fusarium oxysporum*, **(B)** *Macrophomina phaseolina* and **(C)** *Rhizoctonia solani*.

The fungal isolate APR5 showed efficient antagonistic activity against *F. oxysporum*, *M. phaseolina*, and *R. solani* in a dual culture assay. The dual culture plates display the inoculation of endophytic fungus APR5 (on the right side) with the appropriate fungal pathogens (on the left side)*. L. pseudotheobromae* grows and completely covers the colony of pathogen *F. oxysporum* through mycoparasitic activity in 3 days of incubation (**Figure 3A**). The endophyte stops growing when it gets in contact with the pathogens *M. phaseolina* and *R. solani.* Here, both the endophyte and pathogenic fungi compete for the substrate (**Figures 3B, C** **)**. Among the tested three pathogenic fungi, *F. oxysporum* was highly inhibited with an inhibition percentage of 70 ± 0.15%, followed by *R. solani* (66 ± 0.1%) and *M. phaseolina* (54 ± 0.1%).

#### 3.3.2 Volatile metabolites of endophytic fungus

**Figure 4:**
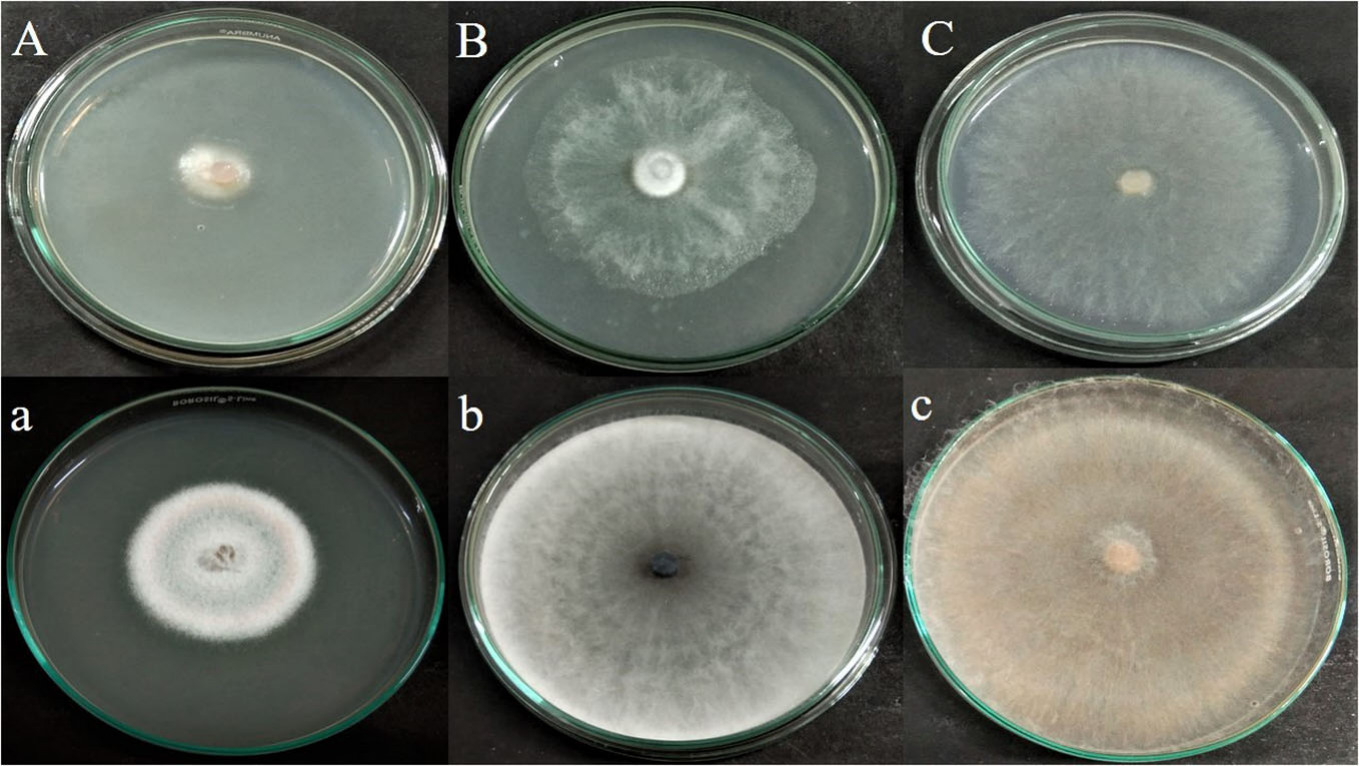
Antagonistic effect of volatile organic compound produced by *Lasiodiplodia pseudotheobromae* APR5, against tested soilborne phytopathogens **(A)** *Fusarium oxysporum*, **(B)** *Macrophomina phaseolina* and **(C)** *Rhizoctonia solani*; (a),(b),(c) are their corresponding control plates.

The antagonistic effect of endophytic fungus APR5 was analyzed by performing a double plate assay. When compared to the control, the development of test phytopathogens was significantly slowed down by the VOCs produced by the fungal endophyte. The pathogenic radical growth in the experimental group was much less than that in the control group (**Figure 4**). The inhibition percentage for *F. oxysporum*, *M. phaseolina*, and *R. solani* was 65 ± 0.1%, 24 ± 0.05%, and 70 ± 0.1% on the seventh day respectively.

#### 3.3.3 Analysis of antagonistic actions using SEM images

**Figure 5:**
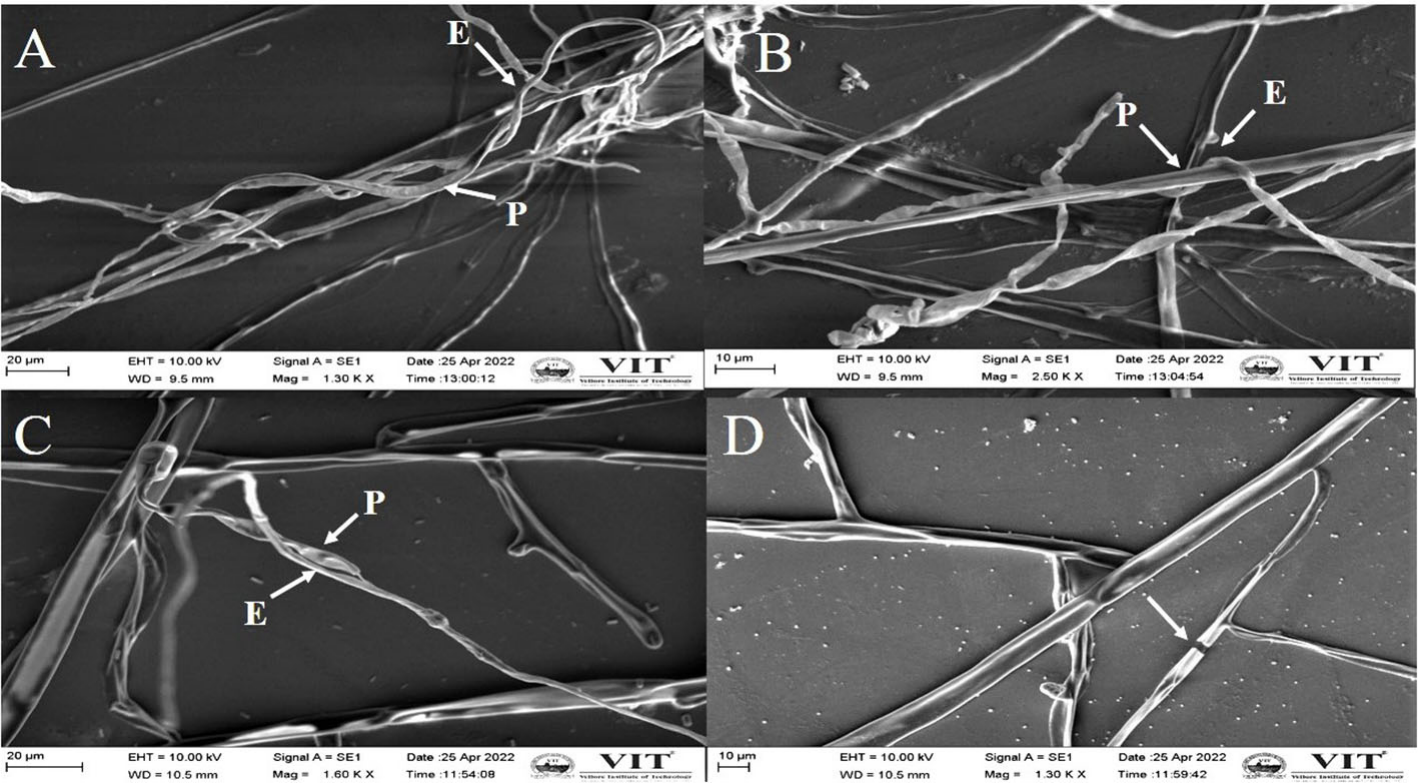
Scanning electron microscopy image demonstrating the morphological changes in the hyphae of *M. phaseolina* **(A)**, **(B)** and *R. solani* **(C, D)**.

The phytopathogenic fungi from the dual culture plate were observed using SEM and it was discovered that the endophytes were responsible for the aberrant morphology of the fungus hyphae. The endophytes induced morphological anomalies in the pathogenic fungal hyphae, according to the SEM findings. The endophyte coiled around the hyphae of pathogenic fungi (**Figures 5A–C** **)**. Shriveling and hyphal disintegration were the morphological alterations observed on the pathogen’s hyphae. The hyphal breakages were observed on the fungal pathogens that are co-cultured with the potent endophyte (**Figure 5D**).

### 3.4 Antifungal bioassay of fungal crude extracts

**Figure 6:**
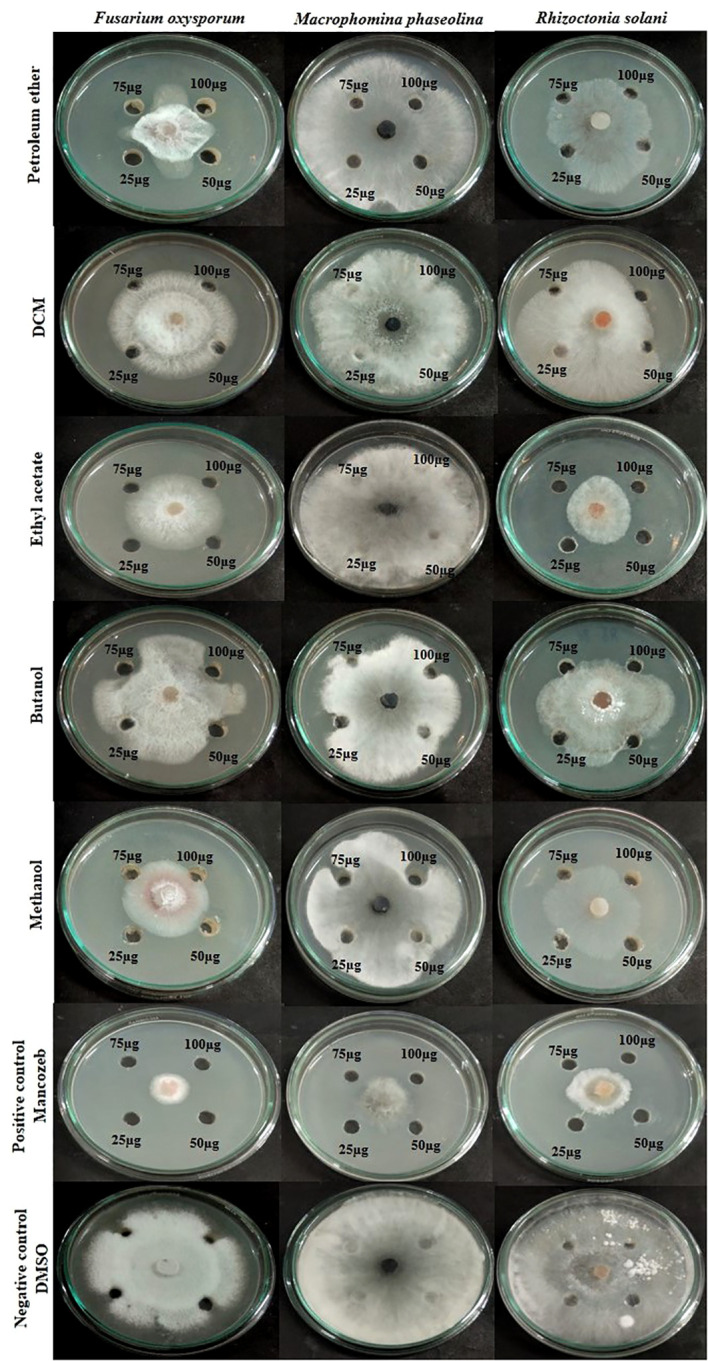
Inhibition of pathogenic fungal mycelial growth induced by different fungal crudes obtained from *Lasiodiplodia pseudotheobromae*.

The impacts of different crude extracts of *L. pseudotheobromae* APR5 on the mycelial growth of soil-borne pathogens (*F. oxysporum*, *M. phaseolina*, and *R. solani)* at different concentrations ranging from 100-25 µg/mL were examined. Significant antifungal activity was observed in the ethyl acetate crude extract against *R. solani* (**Figure 6**). In contrast to the control wells, which were covered with fungal hyphae, the hyphal growth was reduced toward the wells filled with ethyl acetate crude. When compared to the control plate, EA crude inhibited mycelial growth at the rate of 75 ± 0.1%. The organic fractions that were extracted with pet ether suppressed the mycelial growth of *F. oxysporum* with an inhibition percentage of 74 ± 0.05%. The inhibition percentage of all three concentrations of ethyl acetate crude extract was > 50%. The simple linear regression was analyzed using GraphPad Prism 9.5.1. The inhibition rate and log [concentration] value showed a significant linear association based on the outcomes of the toxicity test (R^2^ = 0.9363, p < 0.5). With increasing pet ether extract concentrations, the hyphae’s growth and branching patterns were disturbed, resulting in the aberrant bending of the pathogenic fungal colony. On the other hand, the controls showed normal hyphal development. Results are displayed as the percentage of inhibition in **Table 1**. These findings suggest that *A. paniculata* associated with *L. pseudotheobromae* APR5 have a range of remarkable disease-suppressing properties.

**Table 1:** Antifungal activity of extracts obtained from *Lasiodiplodia pseudotheobromae* isolate of *Andrographis paniculata*.

Pathogen Extract	*Fusarium oxysporum*				*Macrophomina phaseolina*				*Rhizoctonia solani*			
	Concentration µg/mL				Concentration µg/mL				Concentration µg/mL			
	25	50	75	100	25	50	75	100	25	50	75	100
Pet ether	69 ± 0.05	72 ± 0.11	73 ± 0.05	74 ± 0.05	6 ± 0.05	6 ± 0.05	14 ± 0.05	19 ± 0.15	34 ± 0.15	35 ± 0.1	50 ± 0.05	65 ± 0.1
DCM	27 ± 0.07	28 ± 0.1	31 ± 0.05	33 ± 0.1	19 ± 0.1	21 ± 0.1	26 ± 0.1	24 ± 0.1	–	37 ± 0.05	45 ± 0.05	61 ± 0.05
Ethyl acetate	55 ± 0.12	56 ± 0.12	57 ± 0.05	58 ± 0.05	–	7.1 ± 0.1	5 ± 0.05	19 ± 0.1	62 ± 0.15	63 ± 0.1	70 ± 0.1	75 ± 0.1
Butanol	33 ± 0.1	34 ± 0.05	52 ± 0.05	53 ± 0.1	50 ± 0.05	33 ± 0.1	36 ± 0.05	48 ± 0.1	36 ± 0.11	38 ± 0.2	59 ± 0.05	62 ± 0.11
Methanol	54 ± 0.05	55 ± 0.05	66 ± 0.1	69 ± 0.1	63 ± 0.05	62 ± 0.1	26 ± 0.05	24 ± 0.1	47 ± 0.05	51 ± 0.15	58 ± 0.1	64 ± 0.15
Standard	83 ± 0.1	84 ± 0.05	85 ± 0.05	87 ± 0.05	74 ± 0.1	75 ± 0.05	82 ± 0.05	84 ± 0.05	71 ± 0.05	75 ± 0.05	73 ± 0.1	82 ± 0.05

* Values are expressed as inhibition percentage Mean ± SD, n = 3.

‘-’ denotes no antifungal activity.

### 3.5 *In vitro* antifungal activity test

**Figure 7:**
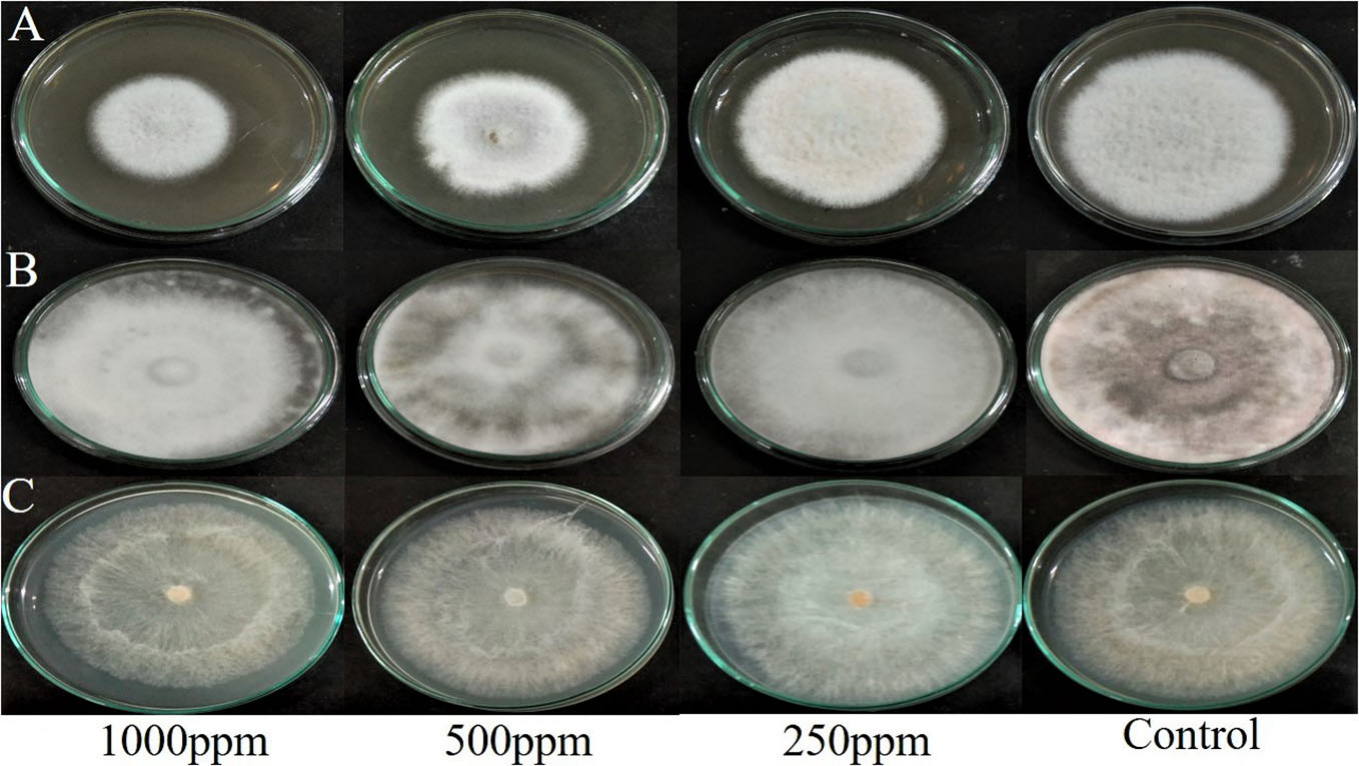
Effect of ethyl acetate crude extract from *Lasiodiplodia pseudotheobromae* on mycelial growth of phytopathogens **(A)** *Fusarium oxysporum*, **(B)** *Macrophomina phaseolina*, and **(C)** *Rhizoctonia solani* at different concentrations (1000 ppm, 500 ppm, 250 ppm, and control DMSO) after 7 days of incubation.

The agar dilution technique was used to evaluate the antifungal activity of EA crude extracts, which showed the highest inhibition of 75 ± 0.1% in the agar diffusion assay. We evaluated various concentrations of fungal crude extracts using the food poisoning method to assess the fungicidal activity. The effectiveness of fungal endophytes in combating phytopathogens was confirmed by the results of the poisoned food approach. The most prominent bioassay for evaluating the efficacy of endophytic fungus against a broad range of diseases is the poisoned food technique (Gupta et al., 2022). The outcomes are shown as the percentage inhibition in radial growth (PIRG) values of pathogens cultured on a PDA medium poisoned with ethyl acetate crude extracts of endophyte. The concentrations of each crude extract that was examined ranged from 0–1000 ppm. The petri dishes were incubated for seven days at room temperature. By monitoring the growth of fungal colonies on all four plates (1,000 ppm, 500 ppm, 250 ppm, and control DMSO), the treatment’s effectiveness was determined (cm) (**Figure 7**). The secondary metabolites present in EA crude extract inhibited the mycelial growth of phytopathogens by reductions in hyphal diameter (**Table 2**). At 1000 ppm, an inhibition percentage of 35 ± 0.05% was observed in the mycelial growth of *F. oxysporum* growth.

**Table 2:** Mycelial growth and inhibition percentage of test phytopathogenic fungi at 1000ppm.

Phytopathogens	Mycelia growth (cm)	Mycelia growth inhibition (%)
*Fusarium oxysporum*	4.8	35 ± 0.05
*Macrophomina phaseolina*	8.5	4 ± 0.07
*Rhizoctonia solani*	7.6	15 ± 0.07

*Values are expressed as inhibition percentage Mean ± SD, n = 3.

### 3.6 Analysis of crude extracts by GC-MS

**Figure 8:**
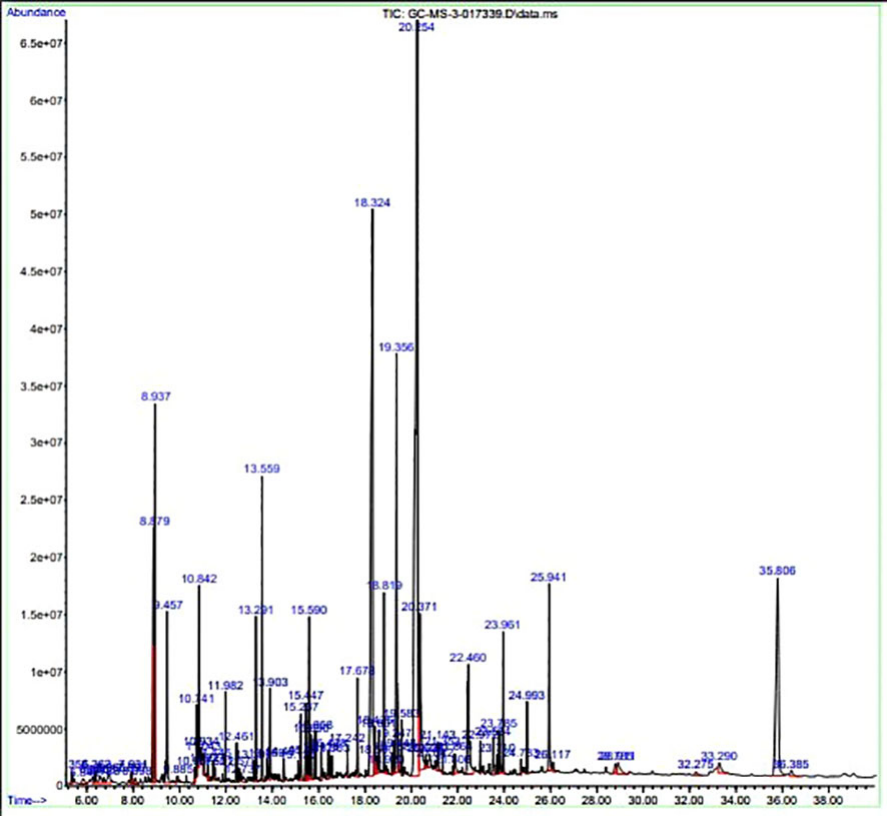
Gas chromatography-mass spectrometry profile of ethyl acetate crude extract from *Lasiodiplodia pseudotheobromae* APR5.

The GC-MS investigation was carried out to identify chemical compounds present in the EA crude extract of *L. pseudotheobromae* APR5, which showed the highest inhibitory effect of 75 ± 0.1% towards *R. solani.* By comparing the mass spectra with the MS spectral database, chemical compounds were identified based on the data of molecular formula, molecular mass, structures, and retention time. The peak area reflected a quantitative percentage of the expected chemical in ethyl acetate crude extract (**Figure 8**). Phenol, erythritol, phenylethyl alcohol, niacin, *t*-butylhydroquinone, 1-octadecene, octadecanoic acid, oleic acid, and *p*-cresol were the chemical compounds with antimicrobial activity identified from the selected crude extract. **Table 3** illustrates a few chemical compounds from ethyl acetate crude with significant biological activity.

**Table 3:** List of chemical compounds in the ethyl acetate crude extract of *Lasiodiplodia pseudotheobromae* APR5.

Name of the compound	RT	Molecular weight	Molecular Formula	Area%	Structure	Biological activity	Reference
Phenol	6.362	94.11	C_6_H_5_OH	0.19	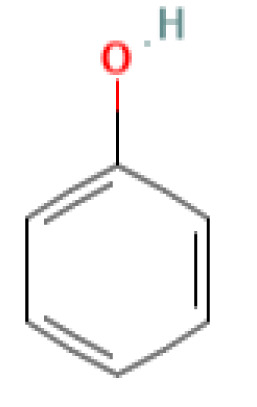	Antimicrobial	(Sabbineni, 2016)
Erythritol	6.723	122.12	C_4_H_10_O_4_	0.12	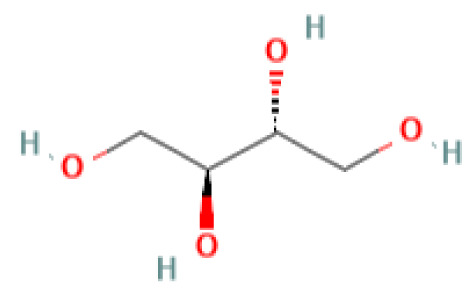	Antimicrobial	(Shimizu et al., 2022)
Pantolactone	8.098	130.14	C_6_H_10_O_3_	0.14	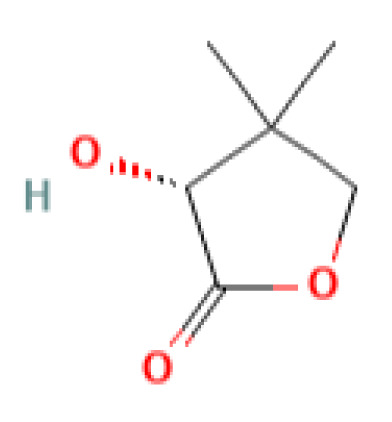	Antiplasmodial	(Baldé et al., 2021)
Phenylethyl alcohol	8.879	122.16	C_8_H_10_O	4.21	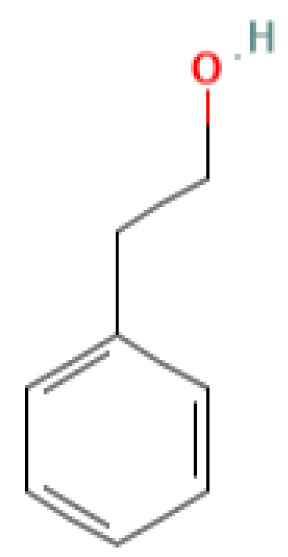	Antimicrobial	(Lilley and Brewer, 1953)
Niacin	10.934	123.11	C_6_H_5_NO_2_	0.13	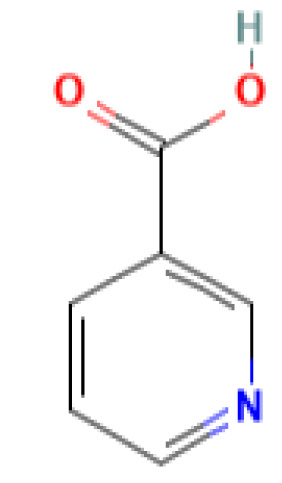	Antimicrobial Antioxidant, Anti-inflammatory, Anticarcinogenic, Antitubercular	(Naglah et al., 2015)
*t*-Butylhydroquinone	15.120	166.22	C_10_H_14_O_2_	0.18	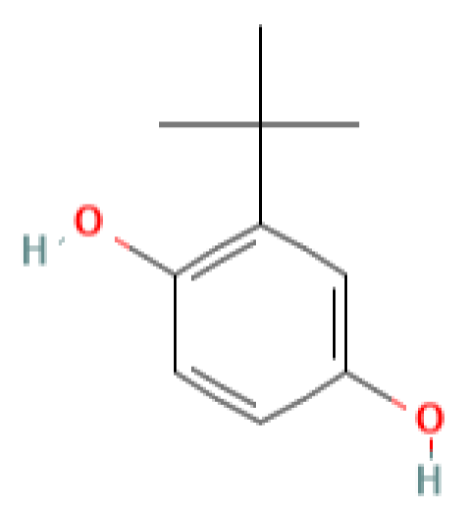	Antimicrobial	(Ooi et al., 2013)
1-Octadecene	15.590	252.5	C_18_H_36_	1.19	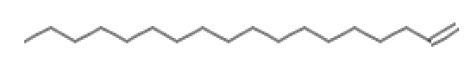	Antimicrobial	(Hameedha et al., 2014)
Octadecanoic acid	20.254	284.5	C_18_H_36_O_2_	25.94	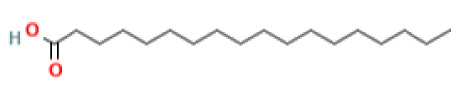	Antimicrobial	(Kima et al., 2016)
Oleic Acid	22.460	282.5	C_18_H_34_O_2_	2.95	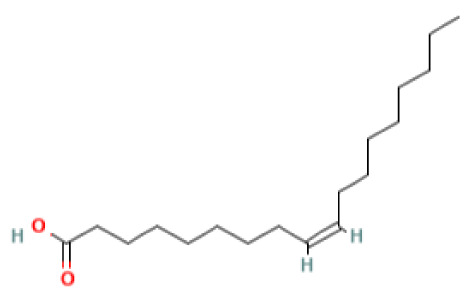	Antimicrobial	(Dilika et al., 2000)
*p*-Cresol	23.785	108.14	C_7_H_8_O	0.39	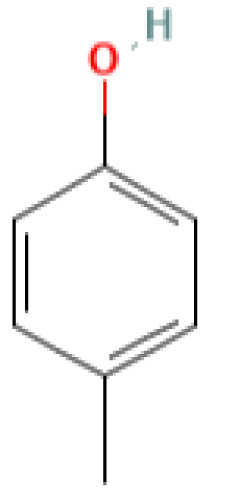	Antimicrobial	(Harrison et al., 2021)

### 3.7 FT-IR Analysis

**Figure 9:**
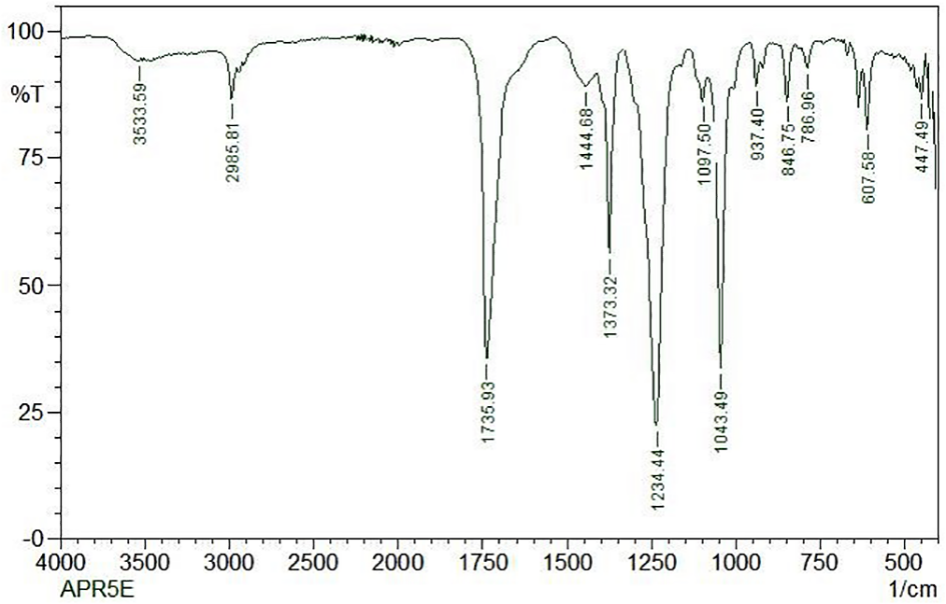
FTIR analysis of ethyl acetate crude extract from *Lasiodiplodia pseudotheobromae* APR5.

FT-IR spectrum of this core exhibited a broad intense peak at 3533.59 cm^-1^ corresponding to the phenolic OH stretching frequency and then the C-H band of alkanes concerning 2985.81 cm^-1^. The presence of the sharp intense bands suggests the adsorption of the capping layer of the nanoparticles corresponds to C = N bond, C–O bond stretch of ether groups, and N = H bond located at the stretching frequency of 1735.93 cm^-1^, 1043.49 cm^-1^, and 1234.44 cm^-1^ respectively. The FTIR spectrum was displayed in **Figure 9**.

### 3.8 Plant growth-promoting traits

**Figure 10:**
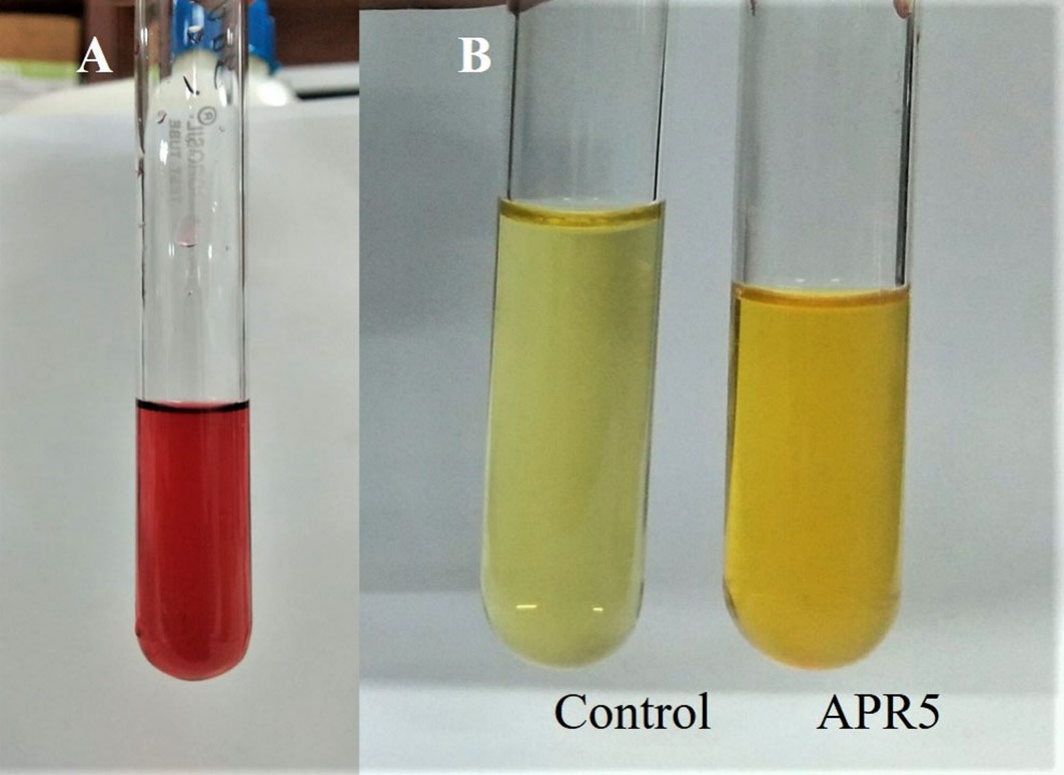
Growth-promoting characteristics of *Lasiodiplodia pseudotheobromae* APR5 **(A)** The production of IAA and **(B)** Siderophore.

The endophyte *L. pseudotheobromae* APR5 was observed to produce IAA which is indicated by the appearance of dark pink color (**Figure 10A**). However, the isolate did not produce HCN. A difference in color intensity (**Figure 10B**) between the test and control samples revealed that the isolate APR5 was a siderophore producer. The lack of brown color development after the addition of Nessler’s reagent confirmed that the ammonia production outcome was not positive. The isolate APR5 was not a phosphate solubilizer, as evidenced by the results of the phosphate solubilization experiment (**Table 4**).

**Table 4:** Growth-promoting traits of *Lasiodiplodia pseudotheobromae* APR5 isolated from *A. paniculata*.

Plant growth-promoting traits	Result
IAA production	+++
HCN production	–
Siderophore production	+
Ammonia production	–
Phosphate solubilization	–

‘+’ indicate positive, ‘-’ indicates no production.

## 4 Discussion

Endophytic fungi offer a lot of promising applications in farming and food production. In recent times, the advancement of new genetic and bioinformatics approaches has enabled the identification of fungal endophytes species with the potential to stimulate the growth of their host plants, due to a range of various processes. The isolation of novel endophytes with significant potential for application in agriculture will be facilitated by studies on microbial diversity in novel plant species as well as in various geographical settings and conditions (Poveda et al., 2021). Without exhibiting any disease symptoms in the hosts, endophytic fungi live inside host plant tissue. Their attachment may be obligatory or facultative and they engage in complicated interactions that include antagonistic behavior and mutualism. The growth of endophytes is severely constrained by plants, but they use a variety of strategies to gradually adapt to their habitats (Ikram et al., 2022). Few investigations have documented the existence of biocontrol agents with the capacity to promote plant growth while simultaneously acting as antagonists against a variety of fungi diseases. Biological control microorganisms have been perceived as a beneficial and ecologically secure alternative to synthetic fungicides for controlling soil-borne diseases (Khan et al., 2021). Several authorized biocontrol agents from the genera *Agrobacterium, Bacillus, Candida, Coniothyrium, Gliocladium, Pseudomonas, Streptomyces*, and *Trichoderma* are widely commercialized (Khan et al., 2021). Therefore, the purpose of this research was to investigate the potential of endophytic fungi to suppress fungal pathogens with a wide host range along with stimulating plant productivity and growth. When used in the field, a biocontrol agent with broad-spectrum antifungal properties has greater prospects than those that are active, particularly against one or two microorganisms (Ali et al., 2020b). Endophytic fungal species from the genera *Curvularia, Chaetomium, Piriformospora, Fusarium, Epicoccum, Trichoderma*, and *Penicillium* are well recognized for increasing the plant host’s resistance towards biotic and abiotic stresses (Rajani et al., 2021; Khan and Javaid, 2022). The synthesis of bioactive compounds, direct competition for nutrients and space with the pathogen, or activation of induced systemic resistance are plausible mechanisms by which *Aspergillus terreus* confers resistance to the host against *Colletotrichum gloeosporioides* (Gupta et al., 2022). VOCs and n-VOCs generated by *Fusarium solani* F4-1007 (endophyte of *Solenostemma arghel*) had the strongest antifungal efficacy, inhibiting *Cochliobolus spicifer* colony formation by 37.27% and 37.1%, respectively. *Penicillium oxalicum* and *Sarocladium kiliense* were the endophytes isolated from the medicinal plant *Aloe dhufarensis* had strong antifungal properties against the pathogenic *Fusarium* sp. and during the VOCs analysis, they revealed the presence of amide, fatty acids, 1,2-diols, fatty acid methyl esters and furfuryl alcohol (Abdel-motaal et al., 2022). In addition to mycoparasitism, VOCs are very crucial for the endophyte *Trichoderma* to combat pathogenic fungi. The development of *Fusarium oxysporum*-CFO, *Sclerotinia sclerotiorum-*TSS and *Sclerotium rolfsii-*CSR were considerably suppressed by endophytic *Trichoderma* sp. in a double-plate experiment (Rajani et al., 2021). *Trichoderma* spp. has drawn a lot of interest for its use in the treatment of *S. rolfsii* due to their exceptional capacity for root colonization, destruction of sclerotia, and generation of antifungal metabolites. Inducing plant defense reactions, producing enzymes that break down cell walls, mycoparasitism, antibiosis, and competition for resources and niches are some of the mechanisms adopted to suppress the development of fungal pathogens (Ali et al., 2020a).

Endophytes associated with medicinal plants have antagonistic behavior toward phytopathogens that cause illness and can produce secondary metabolites that are antioxidant, antimicrobial, and insecticidal (Abdel-motaal et al., 2022). The antibiosis action of strain *Talaromyces* sp. DYM25 prevented the development of *Fusarium equiseti*. The bioactive persistence of filtered broth against *F. equiseti* was initially tested, demonstrating its potential as a bio-control agent across a variety of circumstances including the presence of metal ions, high temperature, an alkaline environment, and UV radiation. In the pot experiment findings, *F. equiseti* induced cucumber wilt, which could be prevented by utilizing the fermentation broth of *Talaromyces* sp. DYM25 (52.9%) (Luo et al., 2021). The diameter of the inhibitory zone clearly showed that the endophytes *Pleosporales* sp., *Phoma* sp., *Cytospora pruinosa*, *Thielavia basicola*, and *Fusarium lateritium* showed the greatest antibiosis towards *Hymenoscyphus fraxineus*. Cytoplasmic extrusions, spiral twists, the formation of torulose hyphae, and excessive lateral branching are the morphophysiological deformations of *H. fraxineus* hyphae, developed under endophyte pressure. The majority of horticulture and crops are the target for the endophyte-based biocontrol techniques that are now being explored. The pathogen *Cronartium ribicola*, which causes the debilitating illness white pine blister rust, was efficiently inhibited by fungal endophytes of *Pinus monticola* (Bila´nski and Kowalski, 2022). *Colletotrichum siamense* isolated from *Piper nigrum* leaves and *Paecilomyces variotii* from *Caralluma acutangula* demonstrated antifungal potential against the widespread pathogen (Poveda et al., 2021). *Phomopsis* sp., an endophytic fungus has attracted a lot of interest in the finding of new biochemically and physiologically effective metabolites and has direct usage in medicine and agricultural biotechnology. Pyrocidines A and B were the antibiotics recently found from the endophyte *Acremonium zeae* of maize and showed considerable antifungal activity against *Fusarium verticillioides* and *Aspergillus flavus* (Yu et al., 2009). 3b-Hydroxy-ergosta-5-ene,3-oxo-ergosta-4,6, 8 and 22-tetraene, 3b, 5a-dihydroxy-6b-acetoxy-ergosta-7,22-diene,and 3b, 5a-dihydroxy-6b-phenylacetyloxy-ergosta-7,22-diene are the antimicrobial steroids from *Colletotrichum* sp., an endophyte of *Artemisia annua*, displayed fungistatic activities towards pathogenic fungi such as *Helminthosporium sativum, Phytophthora capisici, Rhizoctonia cerealis, Gaeumannomyces graminis* var. *tritici*, and *Phytophthora capisici* in the crops. The finding of effective medications or insecticides from endophytes is challenging because most steroid chemicals derived from endophytes have moderate antimicrobial activity. Pestalachloride A and B, two novel antibiotics isolated from endophytic *Pestalotiopsis adusta*, exhibit considerable antifungal efficacy against three plant diseases causing fungal pathogens *Gibberella zeae, Verticillium arboretum* and *Fusarium culmorum*. A group of phenolic acids from *Phoma* sp., of the Guinea plant, inhibits the mycelial growth of *Ralstonia solanacearum* and *Sclerotinia sclerotiorum* (Yu et al., 2009). However, a rising number of publications suggest that the application of endophyte can be reliably used to safeguard forests and ornamental trees (Bila´nski and Kowalski, 2022)

*Lasiodiplodia pseudotheobromae* is a cryptic species that were previously identified as *Lasiodiplodia theobromae* (Alves et al., 2008). In tropical and subtropical areas, *Lasiodiplodia* species are widespread and exist in a range of monocotyledonous, dicotyledonous, and gymnosperm. Lasiodiplodia is a member of the Ascomycota phylum, Dothideomycetes class, Botryosphaeriales order, and Botryosphaeriaceae family, which is composed of 110 species and 17 fungal genera. Members of this family, including the species in the genus, infect a wide spectrum of hosts or live as saprophytes or endophytes inside living tissues (Coutinho et al., 2017). The species has been discovered in Africa, Europe, and Latin America, where it has been found in fruit trees and forests. Similar to *L. theobromae, L. pseudotheobromae* also appears to have a worldwide distribution and a diverse host range. *L. pseudotheobromae* F2 obtained from undamaged *Illigera rhodantha* (Hernandiaceae) flowers exhibited antibacterial activity. Lasiodiplines E from the fungal isolate was effective towards clinical strains such as *Veillonella parvula, Bacteroides vulgates*, *Streptococcus* sp., and *Peptostreptococcus* sp. By modifying bacterial cells and limiting their proliferation, ethyl acetate extract of *L. pseudotheobromae* IBRL OS-64, an endophytic fungus from the leaf of *Ocimum sanctum* was active against Methicillin-resistant *Staphylococcus aureus*. The growth of both Gram-positive and Gram-negative bacteria was significantly suppressed (Jalil and Ibrahim, 2022).

Indole-3-acetic acid (IAA) is a kind of auxin that was associated with plant growth. For the development and growth of shoots and roots, indole acetic acid (IAA) is a crucial chemical substance. Plant growth-promoting compounds like indole acetic acid (IAA) and gibberellins were secreted by endophytic and soil fungi. IAA was more effectively produced by Trichoderma isolate obtained from the rhizosphere region (Syamsia et al., 2015). *Talaromyces* sp. from *Caltha appendiculata* tubers generated an IAA of 7.60 ± 0.32 mg/L on a PDB medium supplied with L-tryptophan (Wang et al., 2022). IAA produced by microorganisms enhances the root surface area and thus improves the uptake of nutrients and water (Ali et al., 2020b). IAA was produced by *Penicillium roqueforti* (CGF 1) in yeast, malt, glucose, and sucrose at concentrations of 36.9 g/mL, 36.0 g/mL, and 35.7 g/mL respectively. IAA levels in *Trichoderma reesei* isolated from *Solanum surattense* were from 40-52 g/mL in sucrose, 39.5 g/mL in yeast and glucose, and 38.0 g/mL in malt extract (Ikram et al., 2022). *Alternaria alternata (Solanum nigrum), Aspergillus awamori (Withenia somnifera), Aspergillus niger (Camellia sinensis), Colletotrichum fructicola (Coffea arabica), Colletotrichum siamense (Piper nigrum), Epicoccum nigrum (Caralluma acutangula), Fusarium tricinctum (Solanum nigrum)* and *Penicillium crustosum (Teucrium polium)* are the IAA producing fungal endophytes. Furthermore, *Aspergillus terreus* obtained from paprika plants is capable of producing IAA in tomato plants, which promotes its growth and inhibits the bacterial speck disease brought on by *Ralstonia solanacearum*, *Pseudomonas syringae* pathovar (pv.) tomato, and *Colletotrichum acutatum*. *Trichoderma harzianum, T. asperellum* and *Paecilomyces formosus* enhance seedling growth, length of shoot and plant biomass in *Capsicum chinense*, whereas *Beauveria brongniartii* from *Carica papaya* improves the diameter of the fruit (Poveda et al., 2021).

*Epicoccum nigrum* isolated from the host plant *Pistacia vera* generates siderophores in the *in vitro* condition. The endophytic fungus *Beauveria brongniartii* can solubilize phosphate and also generates IAA and siderophores on *Capsicum chinense* and *Carica papaya* (Poveda et al., 2021). Plant growth-promoting endophytes actively invade plant tissues and enhances the host plants’ growth and crop yield. The biochemical and physiological metabolism depend heavily on iron. Since the oxidation of ferrous iron and elemental Fe to insoluble ferric iron, cannot support microbial development and the free iron content in the environment is extremely low with the range of 10^−7^ mol (Ikram et al., 2022). The amount of dissolved ferrous iron in calcareous soils is between 10^-10^ to 10^−9^ M which is two to three orders of magnitude less than the amount needed by living things (10^−7^ to 10^−5^ M). The siderophore-mediated iron absorption system used by a few microbes has evolved as a result of environmental constraints and biological necessities. The insoluble ferric iron present in the environment is transported into the cell with the help of siderophores. Various microorganisms synthesize siderophores and combat plant diseases due to this, the bioavailability of iron for pathogens is diminished (Poveda et al., 2021). Therefore, further research into the application of biological control in the management of vegetable diseases will be valuable (Luo et al., 2021). Our study is the first report to reveal *L. pseudotheobromae* as the fungal endophyte from the medicinal plant *A. paniculata.* In addition to providing the foundation for future research and development of new biopesticides from a fungal source. The current investigation established the existence of antifungal inhibitors in crude extracts of the endophytic fungus isolated from *A. paniculata.* To better understand the potential and processes of these natural inhibitors, more research needs to be done to define the bioactive components of the extracts.

## 5 Conclusion

Endophytes can enhance the host plants’ development and resistance to adverse environmental circumstances. Endophytic fungi associated with *A. paniculata* have not been studied in terms of plant-protecting biocontrol agents. Understanding the colonization and function of endophytic fungi found in various regions of medicinal plants is the purpose of this work. For the first time, inhibitors were discovered in crude extracts of endophytic fungi derived from *A. paniculata*, laying the groundwork for future research. To acquire a better knowledge of the capability and actions of natural inhibitors, more research into the bioactive compounds of the extracts should be explored. According to the findings of the present study, the compounds present in the extracts can be used in medicinal applications to safeguard eukaryotic models and plants. However, further research is required to examine all the expenses and advantages of concealing fungal endophytes in a variety of environmental situations to expand the usage and proficiency of endophytes in agriculture.

## Data availability statement

The datasets presented in this study can be found in online repositories. The names of the repository/repositories and accession number(s) can be found in the article/supplementary material.

## Author contributions

The authors confirms sole responsibility for the following: study conception and design, data collection, analysis and interpretation of results, and manuscript preparation. All authors contributed to the article and approved the submitted version.
